# The tree species pool of Amazonian wetland forests: Which species can assemble in periodically waterlogged habitats?

**DOI:** 10.1371/journal.pone.0198130

**Published:** 2018-05-29

**Authors:** Bruno Garcia Luize, José Leonardo Lima Magalhães, Helder Queiroz, Maria Aparecida Lopes, Eduardo Martins Venticinque, Evlyn Márcia Leão de Moraes Novo, Thiago Sanna Freire Silva

**Affiliations:** 1 Programa de Pós-Graduação em Ecologia e Biodiversidade, Instituto de Biociências, Universidade Estadual Paulista (UNESP), Rio Claro, São Paulo, Brazil; 2 Ecosystem Dynamics Observatory, Departamento de Geografia, Instituto de Geociências e Ciências Exatas, Universidade Estadual Paulista (UNESP), Rio Claro, São Paulo, Brazil; 3 Programa de Pós-graduação em Ecologia, Instituto de Ciências Biológicas, Universidade Federal do Pará/Embrapa Amazônia Oriental, Guamá, Belém, Pará, Brazil; 4 Instituto de Desenvolvimento Sustentável Mamirauá, Tefé, Amazonas, Brazil; 5 Departamento de Ecologia, Universidade Federal do Rio Grande do Norte—UFRN, Natal, Rio Grande do Norte, Brazil; 6 Divisão de Sensoriamento Remoto, Instituto Nacional de Pesquisas Espaciais, São José dos Campos, São Paulo, Brazil; Michigan State University, UNITED STATES

## Abstract

We determined the filtered tree species pool of Amazonian wetland forests, based on confirmed occurrence records, to better understand how tree diversity in wetland environments compares to tree diversity in the entire Amazon region. The tree species pool was determined using data from two main sources: 1) a compilation of published tree species lists plus one unpublished list of our own, derived from tree plot inventories and floristic surveys; 2) queries on botanical collections that include Amazonian flora, curated by herbaria and available through the SpeciesLink digital biodiversity database. We applied taxonomic name resolution and determined sample-based species accumulation curves for both datasets, to estimate sampling effort and predict the expected species richness using Chao’s analytical estimators. We report a total of 3 615 valid tree species occurring in Amazonian wetland forests. After surveying almost 70 years of research efforts to inventory the diversity of Amazonian wetland trees, we found that 74% these records were registered in published species lists (2 688 tree species). Tree species richness estimates predicted from either single dataset underestimated the total pooled species richness recorded as occurring in Amazonian wetlands, with only 41% of the species shared by both datasets. The filtered tree species pool of Amazonian wetland forests comprises 53% of the 6 727 tree species taxonomically confirmed for the Amazonian tree flora to date. This large proportion is likely to be the result of significant species interchange among forest habitats within the Amazon region, as well as *in situ* speciation processes due to strong ecological filtering. The provided tree species pool raises the number of tree species previously reported as occurring in Amazonian wetlands by a factor of 3.2.

## Introduction

Knowledge about the biodiversity expected for larger regions, known as the regional species pool [1], is important for inferring evolutionary processes in community assembly [2]. Empirical studies determining the species pool of large regions are central for disentangling the cross-scale processes that shape biodiversity patterns [3] but identifying the species pool of a region is not a trivial task. It requires the accumulation of several biodiversity surveys, well-spaced across the region and covering all possible habitat types. The very definition of species pool as “the set of species able to assemble within a local community” [1,4,5] must be considered before attempting its determination, as the species pool may be defined in terms of a delimited geographic region (i.e. unfiltered pool), or regarding a specific habitat type (i.e. filtered pool) [1,5].

The Amazon encompasses more than one third of all Neotropical plant diversity [6,7], distributed among several habitats with high levels of heterogeneity [8]. Two recently published checklists of the Amazonian flora report overall tree species richness between 6 727 [9] and 11 676 [10] valid species recorded in herbaria, biodiversity repositories and/or inventories, with a predicted richness of c.a. 16 000 tree species [10,11,12] based on inventory observations. The stark difference between checklists comes from a more thorough taxonomic review performed by [9], but regardless of source, both lists can be considered as approximations of the regional unfiltered tree species pool of the Amazon region, in its broadest sense [13].

However, the Amazon region covers more than 7 million square kilometers, spanning 40° of longitude, 25° of latitude, and an elevational gradient of c.a. 6 000 m, and most of the several Amazonian habitats remain poorly sampled [11,14], strongly limiting our knowledge of the true regional species pool. It is unreasonable to expect that all Amazonian tree species are able to occupy every environment, and thus be part of the species pools of all habitats. Thus, to truly understand the processes controlling the assembly and maintenance of Amazon diversity, we must improve our knowledge regarding the filtered species pools [1] of the diverse habitats comprising the Amazon region.

Wetlands have been extensively present in the Amazon since at least the Miocene (30–23 Ma) [15,16], and Pleistocene ocean level oscillations (2.5 Ma) may have strongly influenced their extent and distribution over time [17]. Wetlands currently cover 8.4×10^5^ km^2^ of the Amazon lowlands (c.a. 17% [18]), of which approximately 70% are covered by forests [19]. Total extent may be even higher, comprising up to 30% of the entire Amazon basin, if we consider hydromorphic soils along smaller streams [20–22]. Most Amazonian wetlands show monomodal seasonal fluctuations in water stage and/or water table heights, known as the *flood pulse* [20], which has been inferred to occur at least since the Paleocene (66 Ma) [23].

Hydrological seasonality influences edaphic conditions, leading to hydrological segregation of species niches [24,25] as plants develop the physiological and ecological adaptations necessary to survive several floods and droughts during their lifespan [26–31]. The hydrological regime experienced by each individual tree occurring in the Amazonian wetlands depends on local interactions between basin hydrology and local geomorphology [32], which create strong gradients of flood height and duration, shaping tree species diversification and geographical distribution across scales [22,33–37]. We can thus consider wetlands habitats as environmental filters, selecting individuals and species which can tolerate recurrent inundation and drought during their lifespan (*e*.*g*.: *Hymatanthus* [30]; *Inga* [38]), and it is very likely that Amazonian wetland species have evolved into a particularly filtered species pool.

While most tree diversity studies in the Amazon still focus on upland forests, there has been growing interest in understanding the influence of water-saturated environments on questions related to tree richness [21,37,39], compositional patterns [35,39,40], and phylogenetic diversity [38,41,42]. Available tree species lists for Amazonian wetlands place the eutrophic floodplain (*várzea*) forests as the richest wetland forests in the world, with 918 confirmed tree species [33], and a recent survey of Brazilian Amazonian wetlands raises this number to 1 119 tree species [22], comprising 16% of the 6 727 tree species reported for overall Amazon lowland forests [9]. Furthermore, based on 542 taxa (species and morpho-species), three main biogeographic regions are supported by tree species compositional changes along the Brazilian Amazon river mainstem [35]. It is thus clear that we need a more comprehensive knowledge of the filtered species pool able to colonize these habitats, to better understand the hydrological dimension of niches occupied by Amazonian tree species [24,25] and its role in the assembly and evolution of Amazon rainforests.

Here, we provide the most comprehensive estimate to date of the filtered tree species pool able to assemble in Amazonian wetlands, combining tree species records from herbaria databases and published and unpublished tree species surveys from different types of Amazonian wetland forests. We also discuss the possible role of wetlands in maintaining Amazon tree diversity, and offer a prediction to the expected number of species comprising the total filtered tree species pool that can survive in wetland environments, assessing how it compares to the known Amazon tree flora and predicted basin wide diversity. Finally, we discuss current limitations and best practices for increasing our biogeographical knowledge of the most tree species rich and diverse wetland forests in the world.

## Materials and methods

### Datasets

**Fig 1:**
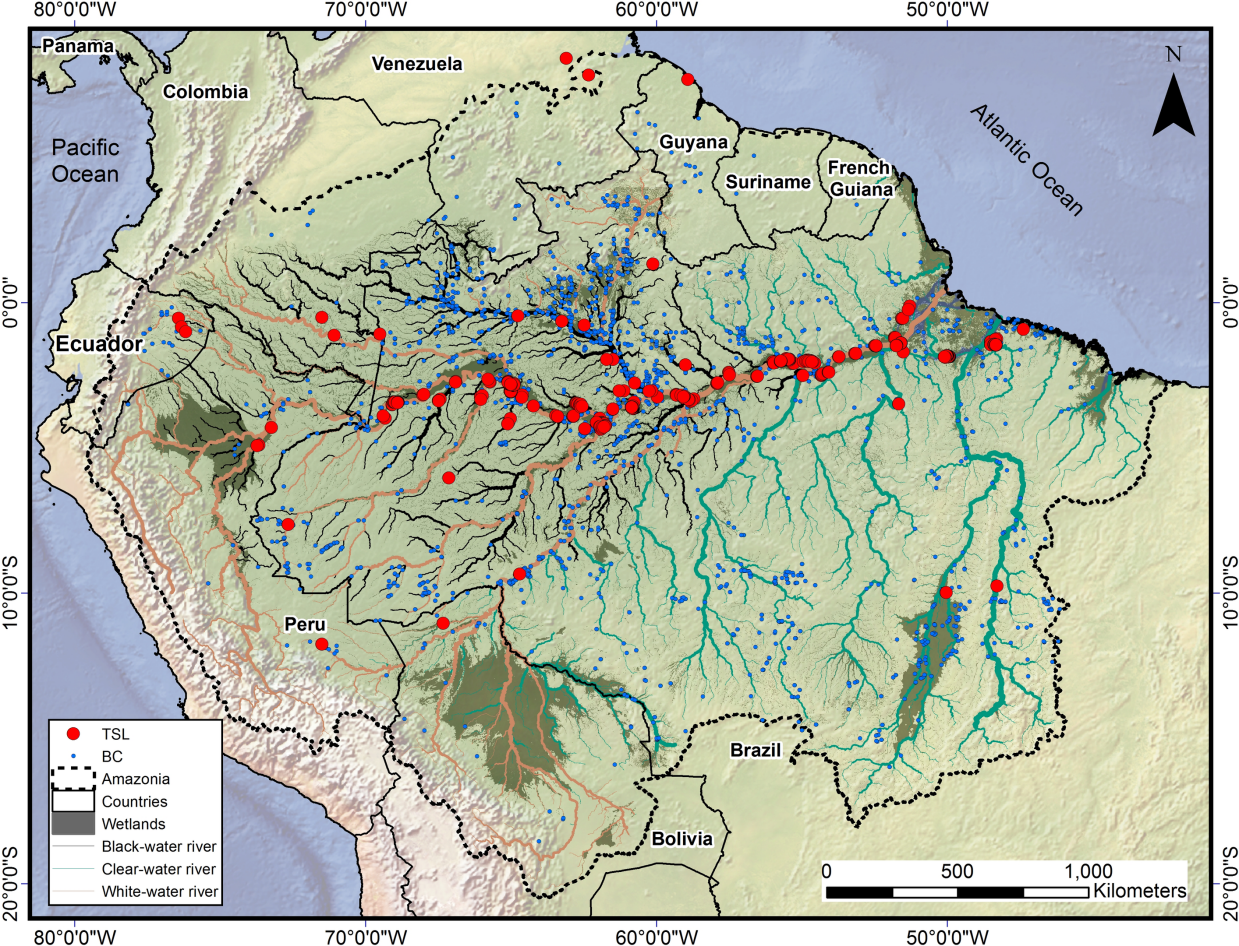
Location of published species lists and herbaria records reporting tree species on Amazonian wetlands forests. The red dots are the location of tree species lists (TSL) from botanical inventories on Amazonian wetlands, blue dots are the voucher specimens from botanical collections (BC). The Amazonia *sensu-latissimo* region is defined in [43], wetland areas were obtained from [44], and the classification of major Amazonian river types is given by [45].

Our first dataset comprises a review of published tree species lists (TSL) from tree plot inventories and/or floristic surveys conducted in Amazonian wetland forests (Fig 1), complemented by one previously unpublished primary inventory of our own (S1 Table). To construct TSL, we only considered studies that reported complete species lists, for any Amazonian wetland type [20].

Our second dataset was built by querying botanical collections (BC) made in Amazonian wetland forests, curated by herbaria (Fig 1, S2 Table) and included in the SpeciesLink digital biodiversity database (http://www.splink.org.br). We queried digitized voucher labels using the following keywords: *“Alagada”*; *“Alagado”*; *“Alagável”*; *“Aluvial”*; “*Alluvial*”; *“Área Úmida”*; *“Brejo”*; *“Chavascal”*; *“Flooded”*; *“Flood”*; *“Floodplain”*; *“Hidromórfico”; “Hydromorphic”*; *“Igapó”*; *“Inundada”*; *“Inundável”*; *“Restinga”*; *“Tahuampa”*; *“Várzea”*. We then merged all botanical records returned for each keyword and filtered these records to include only Angiosperm species and only specimens collected in the Amazonia *sensu-latissimo* region, as defined by [43] (Fig 1).

### Taxonomic standardization

Valid canonical names for species were achieved by performing taxonomic name resolution for both species datasets, using the Taxonomic Name Resolution Service—TNRS V. 4.0 online platform [46]. We set TNRS to perform name resolution without allowing partial matches, and with a minimum match threshold > 0.85. The authority sources consulted were, in order of relevance, tropicos (http://www.tropicos.org) and the plant list (http://theplantlist.org), last updated on August 2015 (for details see: http://tnrs.iplantcollaborative.org). For the TSL dataset, after performing taxonomic name resolution, we filtered the resulting records to remove families known to comprise only non-tree life forms, and we assumed all remaining records after filtering corresponded to tree species. The filtered records from BC dataset were matched to the most recent Amazon tree flora checklist [9], retaining only species names confirmed by taxonomic specialists as valid species names and having a tree life form (i.e. ligneous trunk reaching 10 cm DBH).

### Richness estimation

We used the TSL and BC datasets to build separate species-by-sampling-unit incidence matrices, aggregating incidence by study for TSL, and by year of collection for BC. We used the resulting matrices to assess the chronological order of incidence of each recorded species, building a cumulative species collector’s curve using the ‘vegan’ package [47] and to obtaining the respective sample-based species accumulation curves for each dataset [48]. We then used the sample-based curves to predict the expected species richness if collection efforts were doubled. The inferred and estimated sample-based accumulation curves and predictions of species richness were calculated using rarefaction and extrapolation functions for incidence data provided by [48], using the ‘iNEXT’ package [49]. All analyses were performed in R 3.3.2. [50].

## Results and discussion

### Determining the filtered species pool of Amazonian wetlands

**Fig 2:**
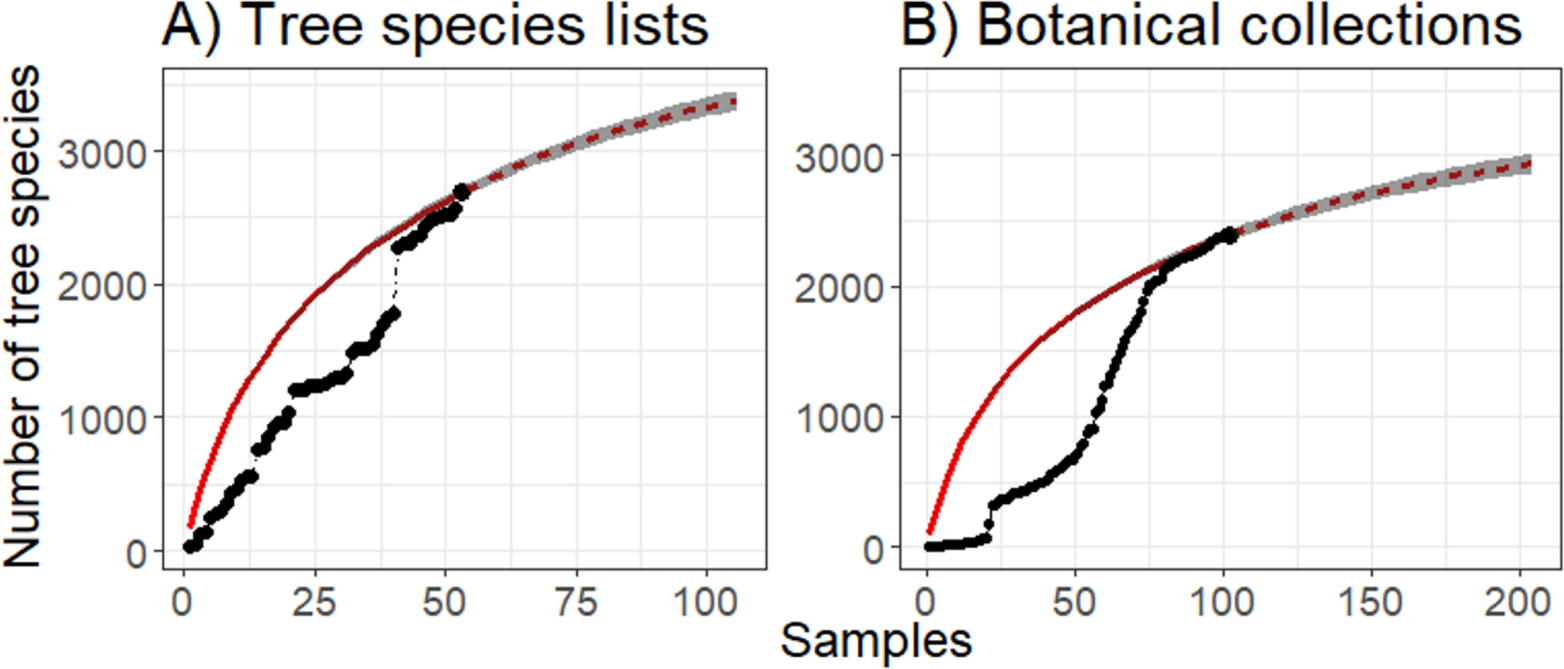
Cumulative collector’s curve and sample-based species accumulation curve for tree species in Amazonian wetlands. (A) Tree species lists (TSL) ordered from 1950 to 2017 (see S1 Table for a list of reviewed studies). (B) Botanical collections (BC) from 1857 to 2016 (see S2 Table for a list of herbaria where records are available). The dots represent the cumulative number of species, the solid red line is the result of random interpolation of these points, and the dashed red line is the predicted number of recorded species with increased effort [48]. The gray area denotes the 95% confidence interval of the estimated curves.

In total, we reviewed 69 studies reporting tree species lists for inventories conduced on Amazonian wetland forests (S1 Table), of which 16 (~ 20%) did not include a complete list of species and could not be added to the TSL dataset. From the 53 studies included in TSL, we recovered 21 446 records comprising 2 688 valid tree species names (S3 Table). From these, we estimate that 3 380 (lower 95% = 3 305, upper 95% = 3 455) tree species would be recorded for Amazon wetland forests if sampling effort was doubled (Fig 2A). Neither the collector’s curve, nor the estimated sample-based species accumulation curve showed signs of reaching an asymptote (Fig 2A), even after almost 70 years of inventories being conducted in Amazonian wetland forests.

We retrieved 231 119 plant occurrence records from the *SpeciesLink* database. After filtering for Angiosperms in the Amazon region, performing taxonomic name resolution and matching against the reference tree species lists, we retained 20 902 records for 2 408 valid tree species names (BC dataset—S2 Table and S3 Table), lower than the observed or expected number of tree species obtained from the TSL dataset. For the BC dataset, we predicted an expected richness of 2 938 tree species (lower 95% = 2 867, upper 95% = 3 009) to be recorded for Amazonian wetland forests if collection efforts were doubled (Fig 2B).

Pooling together the TSL and BC datasets confirmed a total of 3 615 valid tree species, comprising 42 348 records of trees occurring in Amazonian wetland forests (S3 Table), a higher richness than the expected doubling-effort predictions from either isolated dataset. The two datasets shared 1 481 (c.a. 41%) tree species, with 1 207 (c.a. 33%) only recorded by TSL and 927 (c.a. 26%) tree species only recorded by BC.

### Scope and limitations of the determined tree species pool

The determined tree species pool of Amazonian wetland forests comprises 3 615 valid species, encompassing environmental conditions found between diverse wetland types [20]. This is the most comprehensive estimate to date of the Amazonian tree species pool that can survive under extreme hydrological conditions. Although the sampling effort devoted to Amazonian upland forests is currently four times higher than to wetland forests [11, 23], our tree species list represents 53% of all the 6 727 tree species confirmed for the entire Amazon region [9]. Assuming this to be an accurate estimate of the true proportion, Amazonian wetlands could harbor c.a. 8 500 of the 16 000 tree species expected to comprise the total Amazonian tree flora [11].

Most likely, other tree species reported for the Amazon may also occur in hydromorphic environments, but have not yet been recorded in Amazonian wetlands. For instance, the average collection density recovered by us (TSL+BC) is 0.020 records per 100 km^2^ of Amazonian wetlands, when considering the 2.1 million km^2^ estimate of [20], or 0.050 records per 100 km^2^ if considering the more restrictive 840 000 km^2^ mapped by [18]. These sampling densities are three orders of magnitude lower than the observed density of 10 records per 100 km^2^ for Amazonian forests in general [9,51]. For this reason, we also expect that an important portion of tree species occurring in Amazonian wetlands may not be yet known to science. For example, from the 173 tree species discovered in the Amazon during the first decade of the 21^st^ century [52], only 21 (12%) were identified in our estimated species pool, and of these, only six holotype specimens seem to come from vouchers collected in Amazonian wetland habitats. We thus emphasize the dire need for more intensive and comprehensive sampling of the Amazonian wetland environments.

A second limitation of the present list is introduced by the bias towards specific wetland types within the Amazon. Biodiversity assessments in the Amazon and elsewhere are generally biased towards major urban centers and along major rivers or roadways [9,53], and this bias is shown towards inventories of certain types of floodplain forests. The coverage of wetland habitat types and species occurrences recorded in our TSL and BC datasets show, as previously recognized by [23], that eutrophic floodplain forests (*várzeas*) along large “white-water” rivers are the most sampled wetland forest type across the Amazon. Most of the Amazonian human population and major urban centers are adjacent to these areas, and we found the largest densities of botanical records along the Amazonas and Negro river mainstems, near major urban centers with well-established research institutions (*e*.*g*.: Belém, Manaus, Tefé, Iquitos). A much lower record density was observed along the floodplain wetlands of other major Amazon tributaries (*e*.*g*.: Putumayo-Içá; Juruá; Purus and Madeira), or in riparian forests along interfluvial areas of the Amazon lowlands.

A third limitation is that we could not use one in every four (25%) published tree surveys conducted in Amazonian wetland forests, as the authors did not include explicit and complete species lists in the publications. Although the 21^st^ century has seen the rise of collaborative networks, and comprehensive checklists for Neotropical forests provide large amounts of valuable information, we still need a deeper cultural shift among researchers, favoring data sharing and transparency, if we are to improve our combined knowledge of tropical tree biodiversity [54]. It is surprising that the two datasets we investigated shared less than half of the total number of valid tree species recorded, as we would expect complete overlap under an ideal scenario where at least one voucher specimen was deposited for each species recorded in each reviewed inventory (with vouchers properly digitized and made available online in herbaria databases). However, although most published inventory studies claimed to have deposited voucher specimens for their sampled plots, we were unable to find nearly a third of the species reported for inventory plots in the digitized herbaria sources. Very often, easily recognizable species and specimens without fertile structures are not included in voucher collections, creating a “data void” in the herbaria records [51]. Thus, in practice, inventories and isolated botanical collections provide complementary floristic information for assessing tree species diversity. This reinforces the need for including the complete species lists in published inventories and shows that scientists need to keep performing both types of studies if we are to increase our knowledge of the Amazon wetland tree diversity.

Finally, a more comprehensive knowledge of the Amazon wetlands tree species pool can be achieved through efforts in reducing other biological shortfalls (*sensu* [55]). For instance, the uncertainty regarding actual life-form (i.e.: tree) of the recorded plant species (“Raunkiaeran shortfall”), and the lack of voucher determinations and taxonomic reviews for most *herbaria* records (“Linnean shortfall”), resulted in the removal of c.a. 25 000 records and 6 000 species names originally present in the BC dataset after taxonomic standardization and matching to the tree species list of [9]. Furthermore, many samples did not include information on habitat conditions, precluding a detailed assessment of species occurrence by wetland type (*e*.*g*.: *várzea*, *igapó*, *campinas*, *tidal várzeas*). More efforts should be made to ensure forthcoming botanical collections and inventories explicitly include life form and specific habitat conditions, as well as other ecologically relevant information.

### How does the Amazonian wetland species pool compare to the basin-wide species pool?

The tree species pool of Amazon wetlands comprised 104 botanical families distributed into 689 genera, with eleven families having more than 100 tree species each. Leguminosae (578 tree species), Rubiaceae (220 tree species), Annonaceae (182 tree species), Lauraceae (175 tree species), and Myrtaceae (155 tree species) were the most diverse tree families in Amazonian wetland forests, comprising together 36% of the Amazonian wetlands tree species pool. The ten richest families in Amazonian wetlands accounted for 53% of the entire species pool (Table 1).

**Table 1:** Tree species richness for the ten richest botanical families found in Amazonian wetlands compared with their richness ranking according to the Amazon tree flora.

Family	Richness ranking for Amazonian wetlands tree species pool	^1^Richness ranking for Amazonian tree flora	Number of valid tree species in Amazon Wetlands	Number of valid tree species in entire Amazon flora^1^	Percent of species occurring in wetlands (%)
Leguminosae	1	1	578	1 042	55
Rubiaceae	2	5	220	338	65
Annonaceae	3	4	182	388	46
Lauraceae	4	2	175	400	43
Myrtaceae	5	3	155	393	39
Melastomataceae	6	6	136	263	51
Chrysobalanaceae	7	7	132	256	51
Sapotaceae	8	8	128	244	52
Euphorbiaceae	9	11	114	160	71
Moraceae	10	13	112	147	76

^1^Following [9].

Although 69 tree families had half or more of their Amazonian taxa occurring in Amazon wetlands, including some of the richest wetland families (Leguminosae, Euphorbiaceae and Moraceae, Table 1), we did not find any wetland records for 15 families with known occurrence in Amazon forests. Overall, c.a. 51% of the Amazonian tree species within each family occurred in wetland habitats, but there were noticeable differences in rank order and percentage of shared species between the ten richest wetland-occurring families and their respective richness ranking within the overall Amazon flora, as given by [9] (Table 1).

**Fig 3:**
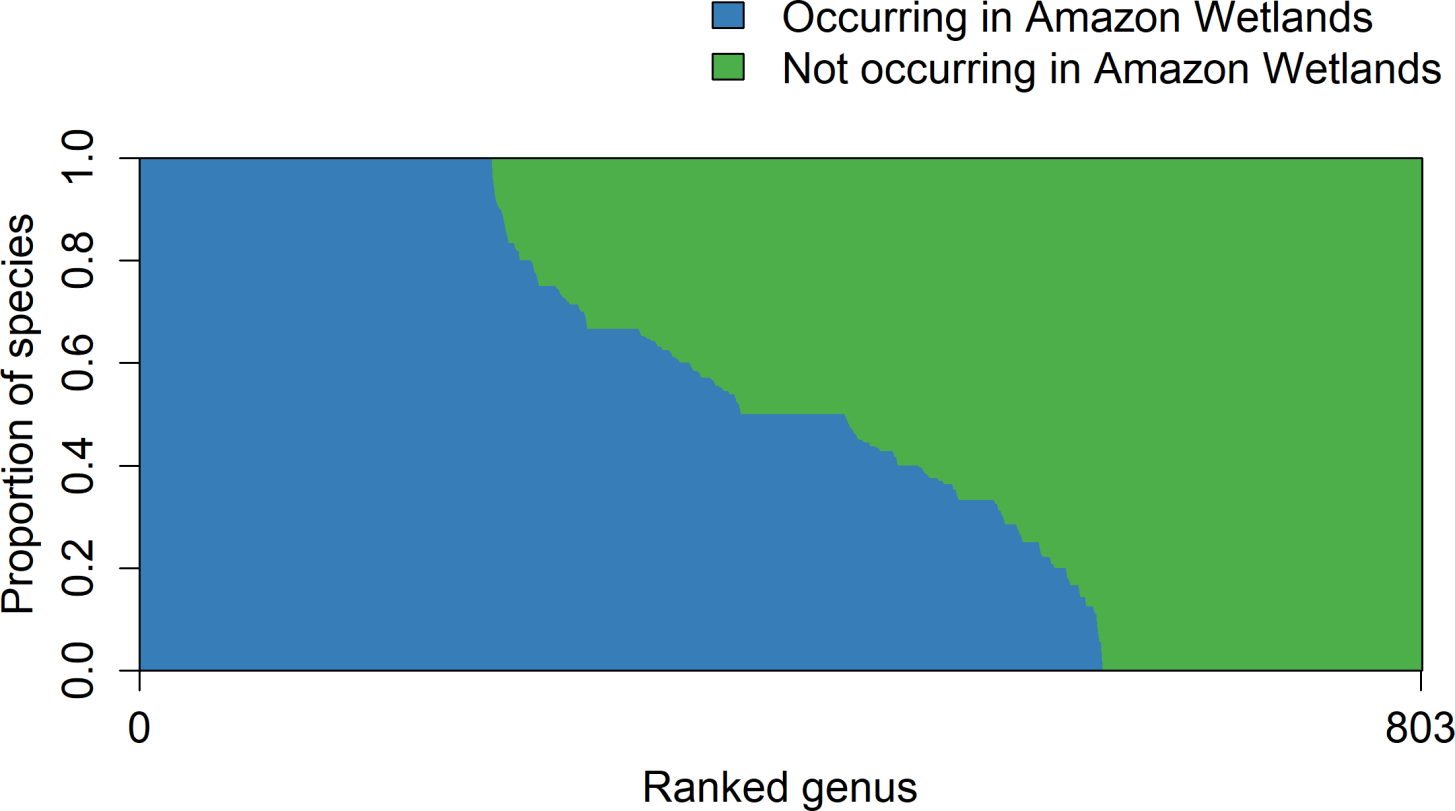
Per-genus proportion of Amazonian tree species occurring and not occurring in wetlands. Proportions are calculated for the 803 genera listed the Amazon tree species checklist [9] and ranked from higher to lower proportion of species on wetlands.

At the genus level, 221 genera in the Amazon tree checklist [9] had all its known species recorded in the Amazon wetlands tree species pool (Fig 3). However, many of these genera (124) had only a single accepted species occurring in the Amazon, with only eight genera having 10 or more known species (max. 26 species). Conversely, 201 genera listed on the Amazon tree checklist [9] had no species recorded in Amazonian wetlands (Fig 3). The richest genus in Amazon wetlands is *Inga* (85 tree species), followed by *Licania* (69 species), *Miconia* (69 species), *Pouteria* (69 species), and *Eugenia* (59 species).

### The ecological and evolutionary role of Amazonian wetlands

The filtered tree species pool for Amazonian wetland forests includes almost all botanical families known to occur in Amazon forests. It is comparable to the 3 389 tree species acknowledged for the entire Brazilian Atlantic Forest [56], one of the most biodiverse Neotropical biomes. One possible explanation for this richness is that, as Amazonian upland and wetland areas are contiguous habitats known to have an interchangeable flora [34,40], we can expect a high degree of lateral migrations among these habitats, with a large proportion of tree species in each lineage reaching and eventually adapting to both flooded and non-flooded forested habitats. Still, different patterns might also be plausible. For instance, the contribution of tree species occurring in Amazonian wetlands to the total diversity of the Amazon-centered genus suggest some taxa have evolved a high degree of *in situ* specialization on wetlands, only then colonizing upland habitats. Despite the high likelihood that a tree species will reach wetland habitats when migrating across the Amazon landscape, many Amazonian tree species do not show preference for flooded habitats; c.a. 64% of the 4 963 tree species recorded in ATDN database [11], with only 68 of the 600 most common tree species occurring in white-water Amazon floodplain forest seeming to be habitat endemics [34]. Assessing phylogenetic history and the relative contribution of each direction of migration to diversification could give us important insight on the origin and evolutionary history of several important taxa in the Amazon tree flora, and the role of strong environmental filtering and hydrological niche specialization in this process, as has been shown for Brazilian C*errado* species in relation to fire disturbance [57].

Growing evidence suggests that it is reasonable to think of a tree species pool comprised by the entire Amazon region [13], but the role of ecological filtering in the assembly of local communities cannot be excluded [58]. The continental dimensions of the Amazon biome and the virtual lack of geographic barriers for plant species across the lowlands implies few dispersal limitations for tree species [13]. New environmental conditions are reached when species expand their distributions, and this floristic interchange between wetland and upland habitats might modulate source-sink population dynamics across marginal habitats. At ecological timescales, source-sink dynamics will affect population regulation and species coexistence [59, 60]; over evolutionary timescales, it will select ecotypes more prone to colonize certain habitats, leading to genetic and morphological differentiation among populations [30, 58, 61]. In this context, although the Amazonian hydrological gradients are more idiosyncratic than the conspicuous and widely discussed temperature gradients along Andean mountain slopes, there is ample evidence for selective pressures acting on the hydrological niche dimension of Amazonian tree species, strongly affecting vegetation development and the distribution of species diversity across the region [11, 39, 62]. Therefore, these lowland hydrological gradients are very likely to have had a strong historical role on tree species diversification, range expansion [34, 38, 42, 63], and local community assembly [37, 39].

## Conclusions

We show that the tree species pool of Amazonian wetlands comprises 53% (3 615) of the confirmed tree species occurring in the overall Amazon, raising previous richness estimates by a factor of 3.2. It is very likely that many of these species will also occur in other forested habitats, or even other Neotropical regions. A large portion of the Neotropical plant diversity is encompassed by Amazon-centered taxa and understanding their evolutionary and ecological histories can improve our knowledge of the development of this hyperdiverse biogeographic realm. Geographical barriers for plant dispersal are mostly absent in the Amazon region, which is instead characterized by a mosaic of habitat types and environmental gradients, including wetland habitats that have been pervasively present since before the Andean uplift. Further studies that can disassemble and then contrast the Amazon tree flora into the filtered species pools associated with each habitat type are necessary to open new avenues for exploring the ecological and geographic distribution of Amazonian tree species, functional types, and lineages, and unveil the relative role of dispersal and environmental filtering on community assembly and on the origins and maintenance of species diversity over time.

## Supporting information

S1 Table Reference list for reviewed tree species lists. (XLSX)

S2 Table List of herbaria available on SpeciesLink that contributed with records. (XLSX)

S3 Table Checklist of the Amazonian wetlands tree species pool. (XLSX)
